# Density-Functional Theory Shows 2*H*‑Tetraphenylporphyrin Prefers Physisorption over Chemical Bonding on Ag(111)

**DOI:** 10.1021/acsomega.5c12411

**Published:** 2026-03-29

**Authors:** Shabnam Naseri, Gustav Johansson, Ghulam Abbas, Muhammad Sajjad, J. Andreas Larsson

**Affiliations:** † Applied Physics, Division of Materials Science, Department of Engineering Sciences and Mathematics, Luleå University of Technology, Laboratorievägen 14, SE-971 87 Luleå, Sweden; ‡ 4566 Linköping University, Department of Physics, Chemistry and Biology, Linköping SE-581 83, Sweden; § Nottingham Ningbo China Beacons of Excellence Research and Innovation Institute, University of Nottingham Ningbo China, Ningbo, Zhejiang 315100, China; ∥ Wallenberg Initiative Material Science for Sustainability, Luleå University of Technology, Laboratorievägen 14, SE-971 87 Luleå, Sweden

## Abstract

Conformational changes upon adsorption can significantly influence a molecule’s behavior at surfaces. In this study, we employ density functional theory (DFT) with the r^2^SCAN+rVV10 functional to investigate the adsorption characteristics of 2*H*-tetraphenylporphyrin (2*H*-TPP) on a Ag(111) surface. We find that 2*H*-TPP physisorbes readily on all adsorption sites, but chemisorption is rare and involves large molecular distortion. The most stable configuration is physisorbed and occurs above the bridge site with an adsorption energy of −6.35 eV, which is 0.95 eV lower in energy than the most stable chemisorbed configuration. Thus, on Ag(111) physisorption is more stable than chemisorption. These findings, supported by electron localization function (ELF) analysis, are contrary to what has been found for the Cu(111) surface, for which chemisorption is the most stable binding. This is further corroborated by potential energy surface calculations using the climate image-nudged elastic band (CI-NEB) method along two reaction pathways, which reveal a 1.2 eV reaction barrier from the physisorbed to the chemisorbed configuration. We have found a barrier of only 0.024 eV between adjacent physisorbed sites, which is large enough to render it immobilized at room temperature. The results provide compelling evidence that 2*H*-TPP physisorbs on Ag(111) and will not chemically bond under normal circumstances.

## Graphical abstract

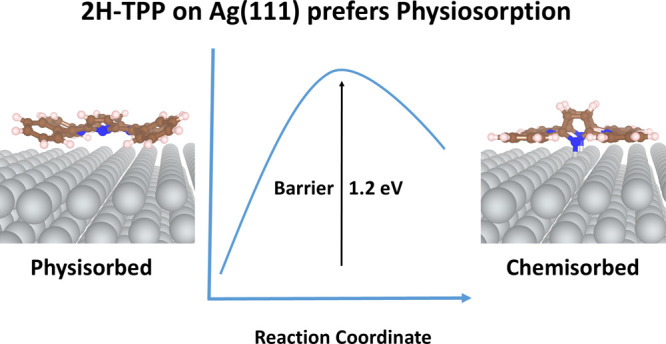

## 1 Introduction

The study of porphyrin molecules on surfaces has gathered much interest in recent years due to their broad applications in various fields such as photonics, molecular electronics,^1^ and solar cells.^2^ Porphyrins, metalloporphyrins, and their derivatives are relatively nontoxic and thanks to their exceptional, reversible oxidation and reduction chemistry, many porphyrins have the potential to be used as wires, switches, transistors, junctions, and photodiodes.^1^ Several investigations have shown the potential of porphyrin molecules in molecular electronic devices^3–5^ and solar cell applications, highlighting how the molecular structure affects the electronic characteristics and how surface chemistry, attachment, and orientation influence their performance.^6^

The porphyrin core macrocycle can host various metal atoms, in metalloporphyrins,^7^ introducing additional functionalities such as sensing^8,9^ or magnetic properties that can be used in storage media applications.^10–12^ Furthermore, their properties can be changed by alterations within their macrocycle giving rise to porphyrin derivatives such as the tetraphenylporphyrin (TPP).^13–20^ It should be noted that porphyrins generally have a flat and robust structure, enabling them to resist thermal preparation processes.^21,22^ The flat conformation affects the molecular behavior, leading to unique ligation or self-assembly characteristics.^13^ The TPP molecule typically adopts a saddle-shaped conformation when adsorbed onto a metal surface.^13–20^ However, recent experimental studies have provided direct evidence of both saddle and inverted conformations for porphyrins adsorbed on metal surfaces, demonstrating unique spectral fingerprints that confirm these structural motifs.^23^ As a result, the development of technical applications relies heavily on the specific properties of individual molecules at the molecular or atomic scale,^24^ with structural flexibility being a crucial factor in achieving conformational differences that influence molecular performance.^6^

The unique properties of porphyrins make them promising building blocks for assembling functional layers and nanostructures on substrates, leading to new possibilities in sensor development and nanoscale optical and magnetic materials.^25,26^ The self-assembly and performance of porphyrins depend on three key factors: 1) the porphyrin core macrocycle, which provides active sites for forming porphyrin–ligand complexes,^27,28^ 2) the meso-substituents, which control the molecule’s behavior and serve as building blocks for metal–organic networks in solid state chemistry^29^ and 2D molecular structures,^30,31^ and 3) the flexibility of the porphyrin nucleus, which allows for structural adaptation to its local environment^32,33^ and manipulation.^5,34–36^

There exist numerous examples of porphyrins forming well-ordered 2D molecular domains. It was shown that annealing multilayer free-based tetraphenylporphyrin (2*H*-TPP) on Ag(111) to 550 K results in a saturated monolayer and leads to intramolecular cyclodehydrogenation, transforming the molecule into a flat porphyrin.^37,38^ The 2D molecular domains are typically formed through physisorption, which was found for 2HTPyP on Ag(111)^25^ and 2*H*-TPP on Ag(111).^39^ The self-assembly of *x*-TPP (*x* = Fe, Co, 2H) on Ag(111) showed physisorption at various coverages.^40^ It should be noted that porphyrins can bind to metals via both physisorption as well as chemisorption. This was shown for Br_4_TPP, which self-assembles on Cu(111) at low temperatures, with both physisorption and chemisorption possible.^40,41^

In particular, adsorption studies on small silver clusters have provided further insight into the nature of physisorption and chemisorption on silver, which is relevant when extrapolating behavior to extended surfaces like Ag(111).^42^

The various adsorption possibilities are particularly evident for the 2*H*-TPP molecule, which has been studied extensively. DFT calculations have shown that 2*H*-TPP can bind to Cu(111) via both chemisorption and physisorption.^23,43^ Further investigation of the 2*H*-TPP molecule on the Ag(111) substrate revealed that the macrocycle substrate adopts a saddle-shaped structure, while the phenyl legs show a tilt angle of 50–55° relative to the surface.^44^ Other studies have indicated intermolecular repulsion for 2*H*-TPP on Cu(111)^13,18,20,45,46^ and attractive interactions on Ag(111).^40,41,45–52^ Hence, despite extensive experimental and theoretical efforts, the detailed mechanisms underlying 2*H*-TPP adsorption on Ag(111) remain under debate, particularly the interplay between physisorption and chemisorption and the role of conformational flexibility in stabilizing different adsorption states. Furthermore, earlier investigations have shown that the molecule can adopt multiple metastable adsorption states, attributed to its structural flexibility and the homogeneity of the Ag(111) surface.^53^ Moreover, previous DFT studies often lacked accurate treatment of nonlocal dispersion forces, which are crucial for describing the weak interactions dominating such systems. In fact, recent studies have shown that standard DFT approaches often fail to capture weak interactions accurately, especially in systems involving aromatic molecules adsorbed on metal surfaces. The incorporation of van der Waals density functionals (vdW-DF) has significantly improved the modeling of adsorption energies and geometries in such systems.^2–4^ In this work, we address the open issues of the adsorption behavior of 2*H*-TPP on Ag(111) by employing the state-of-the-art r^2^SCAN+rVV10 functional within the framework of DFT. By analyzing adsorption geometries, energetic profiles, electron localization functions (ELFs), and diffusion barriers via climbing image-nudged elastic band (CI-NEB) calculations, we provide new insight into the balance of covalent and van der Waals interactions. Our findings reveal that physisorption is energetically favored and that molecular conformation plays a key role in stabilizing the adsorbed state, challenging previous assumptions of predominant chemisorption. These results offer a refined understanding of molecule–surface interactions in π-conjugated systems and contribute to the rational design of surface-supported molecular architectures.

## 2 Computational Details

All calculations were performed using the Vienna Ab initio Simulation Package (VASP)^54,55^ and the projector augmented wave (PAW) method.^56,57^ The plane-wave energy cutoff was set to 460 eV, and a Gaussian smearing of 0.05 eV for the initial electronic occupations was used. The calculations were performed using the r^2^SCAN-rVV10 meta-GGA dispersion functional.^58,59^ Separate calculations were performed with the optB86b-vdWDF dispersion functional^60^ as well as with the semiempirical correction for dispersion, PBE-D3 with Becke–Jonson damping, proposed by Grimme.^61^ A 1 × 1 × 1 Monkhorst–Pack^62^ k-mesh and a 20 Å vacuum in the out-of-plane direction were used to avoid unnecessary interaction due to periodic boundary conditions. The model was constructed as a 12 × 12 × 1 supercell of a four-layered Ag(111) slab containing 576 Ag atoms in total. The 2*H*-TPP (C_44_H_30_N_4_) molecule with 78 atoms was placed parallel to the surface. All atoms of the 2HTPP/Ag(111) system were allowed to fully relax until the Hellmann–Feynman forces were below 1 meV/Å for all atoms. The Virtual NanoLab software^63^ was used to set up the simulation geometries, and the visualization of the electron localization function (ELF) was carried out with VESTA.^64^ The reaction barrier was calculated using the climbing image-nudged elastic band (CI-NEB) method.^65,66^ Seven intermediate images were generated to simulate the two separate reaction paths of physisorbed to chemisorbed and physisorbed to adjacent physisorbed configuration of 2*H*-TPP. Due to the high computational cost of CI-NEB simulations for such a large system, calculations were carried out using the PBE-D3 approach, with relaxed force convergence criteria set to 0.04 eV Å^–1^.

## 3 Results and Discussion

The adsorption of 2*H*-TPP on Ag(111) is a complex matter with several degrees of freedom involved (Figure 1). In addition, previous studies indicate that the molecule exhibits numerous metastable adsorption configurations due to their inherent flexibility and the relatively uniform nature of the Ag(111) surface.^53^ Most metal-free porphyrins, as observed in both theoretical and experimental studies, are bound through either physical binding or chemical bonding.^1–5,21,22^ These porphyrins typically adopt a “saddle” conformation or, in some cases, an “inverted” structure. In contrast, metalloporphyrins generally form chemical bonds via their central metal atom.^25^

**Figure 1:**
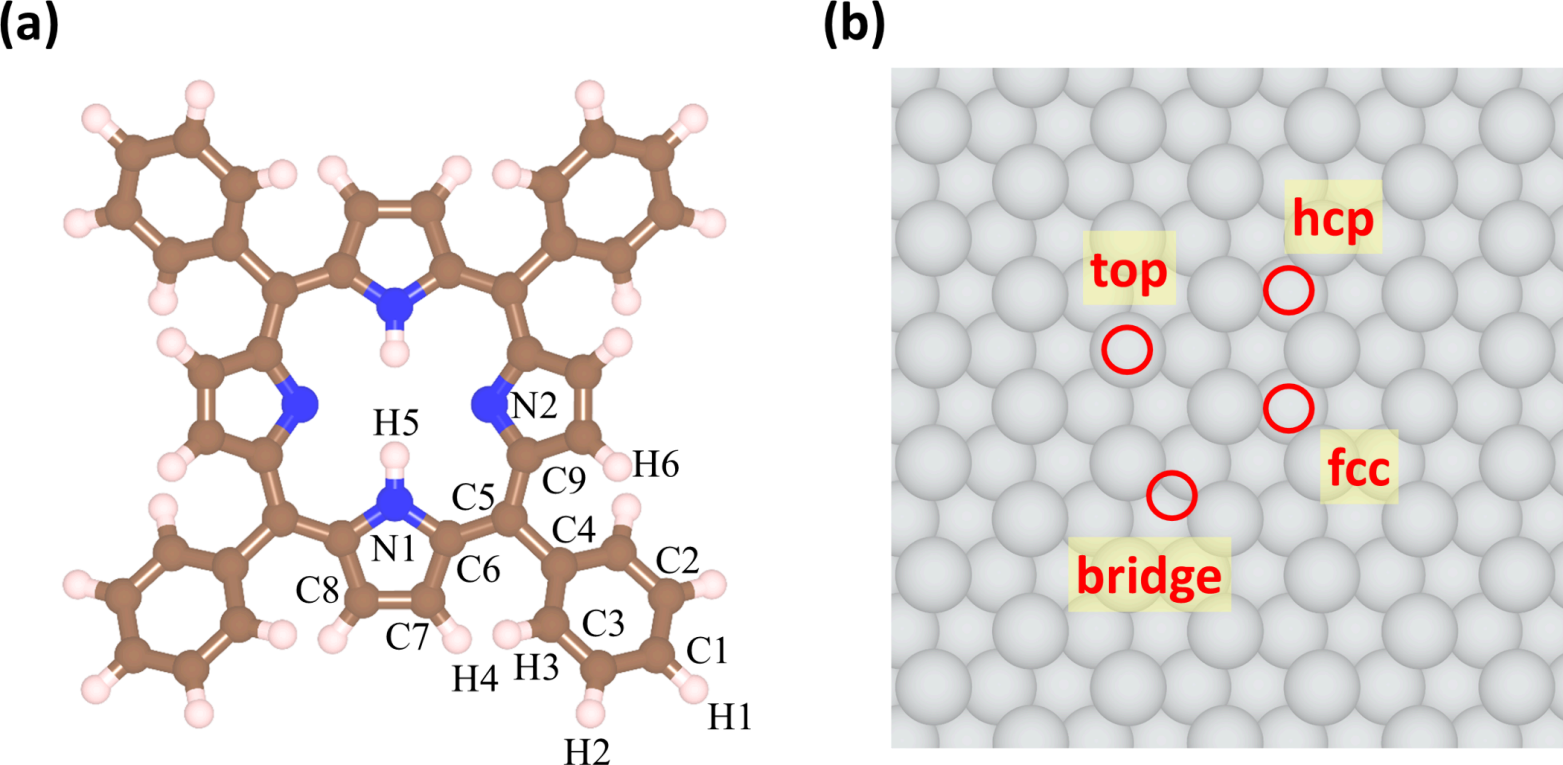
(a) The structure of the free-base tetraphenylporphyrin (2*H*-TPP) molecule. Brown, blue, and white spheres correspond to carbon, nitrogen, and hydrogen atoms, respectively. (b) A schematic diagram of the Ag(111) slab with different adsorption sites labeled. The gray spheres depict silver atoms.

The r^2^SCAN+rVV10 functional was chosen for this study because it offers a reliable and efficient balance between accuracy and computational cost, particularly for systems combining both metallic and molecular components, where both covalent interactions and van der Waals (vdW) forces are significant. The r^2^SCAN meta-GGA functional is a numerically stable and computationally efficient refinement of the SCAN functional, known for its improved treatment of intermediate-range correlation effects and an accurate description of diverse bonding environments. When augmented with the rVV10 nonlocal correlation functional, it provides an enhanced description of dispersion interactions, which are crucial in accurately modeling the adsorption behavior of large π-conjugated systems like 2*H*-TPP on metal surfaces. This approach allows for capturing both chemisorption characteristics and vdW contributions that dictate the adsorption geometry and energetics of the molecule on the Ag(111) substrate.

To identify the most stable adsorbate geometry, the molecule was placed at all high-symmetry sites of the metal (111) surface, i.e., top, bridge, fcc, and hcp as shown in Figure 1. For each site, three different in-plane orientations (0, 45, and 90 relative to the *a*-axis of the surface) were considered to ensure a thorough sampling of the configurational space. With these as starting positions, the structures were fully relaxed as described above until they could be discarded either as they relaxed into the same conformation or were too energetically unfavorable. The stability is evaluated using the binding energy, which is defined as

$$E_{B}=E_{\mathrm{sys}}-E_{\mathrm{slab}}-E_{\mathrm{mol}}$$ 1

where *E*_slab_ is the total energy of the clean slab, *E*_mol_ is the total energy of the isolated gas-phase molecule (see Figure 1), and *E*_sys_ is the energy of the combined system, i.e., silver slab and the molecule. The most stable geometry on the substrate was found to be oriented such that the iminic nitrogens (N) were centered on fcc sites along the *a*-axis, shown in Figure 2a–c.

**Figure 2:**
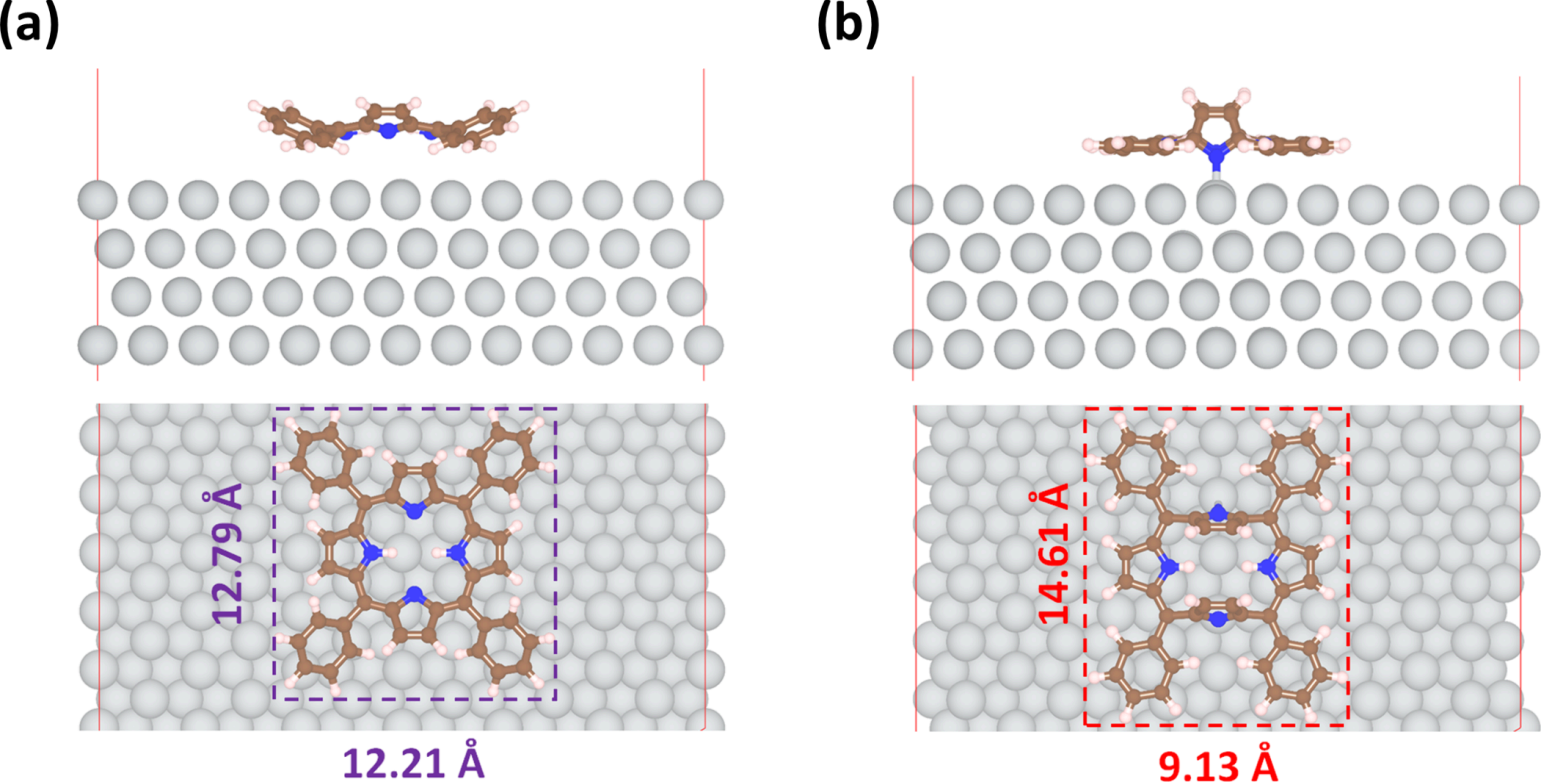
The r^2^SCAN+rVV10 relaxed geometries of 2*H*-TPP on the Ag(111) surface: (a) physisorbed 2*H*-TPP and (b) chemisorbed 2*H*-TPP in side and top views, respectively. The gray, blue, and white spheres represent Ag, C, N, and H atoms, respectively.

This configuration is found to be physisorbed, as will be extensively discussed later, with an adsorption energy of −6.35 eV (−6.25 with DFT-D3). The binding energy is considerably larger than for 2*H*-TPP physisorbed on Cu(111) (−4.49 eV with DFT-D3 ^43^). The orientation is also different with the iminic N on the fcc and hcp sites for Cu(111). As seen in Figure 2a, the phenyl legs exhibit a tilt relative to the surface in the physisorbed state, whereas in the free molecule they adopt an even more pronounced tilt, with the pyrrole rings oriented more vertically. This suggests that the reduction in the physical interaction between the legs and the surface is compensated by a stronger binding through the molecular core and reduced steric repulsion. This is what is usually termed a saddle-like conformation. The binding conformation is roughly square (0.58 Å difference between the sides) and is very similar to what is found for the Cu(111) substrate^43^

It should also be mentioned that the adsorption energy of 2*H*-TPP physisorbed on Ag(111) is considerably stronger than what was previously calculated using nonperiodic, dispersion corrected DFT calculations^67^ and what was found for adsorption on the terrace of a Ag(111) step-edge (0.44 eV) using GGA-HCTH calculations.^68^

In addition, close to the stable physisorbed configuration, a chemisorbed conformation was also identified with the iminic nitrogens (N) on top sites, oriented along the *a*-axis, as shown in Figure 2d–f. This conformation differs significantly from the physisorbed one: (i) the shape of the molecule is rectangular with a 5.48 Å difference in side lengths; (ii) the outer part of the molecule adopts a flatter geometry, with the phenyl rings oriented nearly horizontally; (iii) the core of the molecule adopts an inverted structure, with the iminic pyrrol groups standing vertically to accommodate the formation of covalent N–Ag bonds with the surface.

It is also slightly different to what was found for 2*H*-TPP chemisorbed on Cu(111)^43^ with its smaller lattice parameter supporting a more relaxed conformation.

In contrast to the typical situation, the binding energy for this chemisorbed configuration is weaker (−5.40 eV). Although covalent bonding contributes to stabilization via electron sharing, the associated deformation energy cost is evidently higher. The substantial structural distortion of 2*H*-TPP leads to a rectangular shape that is elongated along the direction of the chemically bonded nitrogen atoms and a compressed flat, perpendicular to iminic pyrrole rings (Figure 2b). This contrasts with the more square-like footprint shape observed in the physisorbed state (Figure 2a). Table 1 provides an overview of the binding energies of the two stable configurations calculated using r^2^SCAN+rVV10, as well as with optB86b-vDWDF2 and PBE-D3. While the adsorption energies are seen to be quantitatively different for the different methods (particularly the optB86b-vDWDF2 results), they are qualitatively similar. The geometries of the conformations are also very similar, which is shown in the SI and summarized below.

**Table 1:** Binding Energies for Physisorbed and Chemisorbed Configurations of 2HTPP on Ag(111) and the Corresponding Energy Differences Calculated Using Three Different DFT Methods: r^2^SCAN+rVV10, optB86b-vDWDF2, and PBE-D3

	*E*_B_ (eV)		
Method	Physisorbed	Chemisorbed	Δ*E*_B_ (eV)
r^2^SCAN+rVV10	–6.3478	–5.3974	0.9504
optB86b-vDWDF2	–5.8069	–4.8907	0.9162
PBE-D3	–6.2462	–5.4243	0.8219

Thus, the physisorbed configuration is the most stable geometry on the substrate, and it is 950 meV more stable than the chemisorbed configuration.

Table 2 presents the most significant bond length variations observed between the physisorbed and chemisorbed configurations compared to the gas phase (a complete list of bond lengths is available in Table S1 of the Supporting Information). The molecules appear different in the physisorbed and chemisorbed configurations. For example, the C4–C5 and C9–C10 bond lengths increase compared to the free molecule in the physisorbed configuration but decrease in the chemisorbed one. In the chemisorbed case, the phenyl groups lie flat against the surface, whereas they are tilted in the physisorbed configuration. The tilting of the phenyl groups causes the C4–C5 bond to be longer in the physisorbed configuration compared to the gas phase, while in the flat chemisorbed configuration it becomes shorter. Additionally, the atoms move even closer to the silver substrate when the iminic pyrrole groups are oriented vertically in the chemisorbed configuration. This causes the C5–C9 and C6–C7 bond lengths to increase compared to the free molecule, while they are decreased in the physisorbed configuration. The C3–C4 bond length remains the same for the free molecule and the physisorbed configuration but increases in the chemisorbed one. The C9–N2 bond length increases in both the physisorbed and chemisorbed configurations compared with the free molecule.

**Table 2:** Comparison of Bond Lengths (in Å) of 2*H*-TPP in the Gas Phase and When Either Physisorbed or Chemisorbed on Ag(111)^a^

Bond	Free molecule	Physisorbed	Chemisorbed
C3–C4	1.410 (1.409, 1.405)	1.410 (1.410, 1.405)	1.420 (1.421, 1.417)
C4–C5	1.474 (1.474, 1.470)	1.482 (1.483, 1.479)	1.461 (1.465, 1.460)
C5–C9	1.438 (1.437, 1.433)	1.419 (1.420, 1.414)	1.485 (1.489, 1.487)
C9–C10	1.452 (1.451, 1.446)	1.456 (1.456, 1.450)	1.405 (1.403, 1.395)
C9–N2	1.368 (1.368, 1.363)	1.374 (1.374, 1.369)	1.388 (1.388, 1.386)
C6–C7	1.439 (1.438, 1.433)	1.435 (1.434, 1.429)	1.453 (1.454, 1.449)

a The calculations are performed using the r^2^SCAN+rVV10 (optB86b-vDWDF, PBE-D3) functional. The numbering of atoms is based on Figure 1.

The DFT-calculated geometries reveal that vdW forces play a dominant role in molecular adsorption. Like earlier investigations of large planar molecules,^69^ the vdW interactions were found to be crucial.

Table 3 provides the distance between the 2*H*-TPP molecule and the surface. A comparison of these distances for the two adsorption configurations quantifies the height difference between physisorption and chemisorption. The average heights of the carbon and nitrogen atoms from the top Ag layer for r^2^SCAN+rVV10 (first column in Table 3) show that the physisorbed configuration is positioned 0.576 Å higher than the chemisorbed one, indicating a measurable difference.

**Table 3:** Heights and the Shortest Distances to Ag for 2*H*-TPP Physisorbed or Chemisorbed on Ag(111) in Å for r^2^SCAN+rVV10^a^

			*d*(N)			*h*(N)	
	h̲ (C/N)	*d*(C)	aminic	iminic	*h*(C)	aminic	iminic
physisorbed	3.441	3.126	3.613	3.471	2.831	3.306	3.314
chemisorbed	2.865	2.783	3.043	2.283	2.741	2.907	2.134

a *h*(C/N) is the average height of the C and N atoms to the Ag surface; *h*(C) and *h*(N) are the minimum heights of a C atom and N atom, respectively, to the Ag surface; *d*(C) and *d*(N) are the shortest distances between a Ag atom and a C and N atom, respectively.

The chemisorbed distance is similar to what was found for 2*H*-TPP on Cu(111) and the physisorbed distance is slightly larger, around 0.2Å (see ref 43). For the physisorbed molecule, the average distance of the aminic and iminic nitrogens (3.31 Å) is close to the results of the nonperiodic DFT calculation on 2*H*-TPP on Ag(111) in ref 67 (3.30 Å). It should be noted that similar computational work complemented with experimental results was done for Au-TPP on Au(111) showing that the molecule preferably adsorbs in a top position. While the binding energy is of the same order as our result (5.55 eV), the distance is significantly longer (4.15 Å).^70^

The key differences become evident in the shortest bond distances, particularly between C–Ag and N–Ag, which distinguish physical binding from chemical bonding. In the chemisorbed configuration, the C–Ag bonds measure 2.783 Å, whereas, in the physisorbed one, the shortest C–Ag distance is 3.126 Å. For the iminic nitrogen atoms, the chemisorbed configuration exhibits N–Ag bonds of 2.283 Å, while, in the physisorbed one, the shortest N–Ag separation is 3.471 Å.

Distinguishing between physisorption and chemisorption is not straightforward based only on adsorption heights and distances, particularly for molecular adsorption on metal surfaces. However, these two adsorption modes exhibit distinct electron density characteristics: physical binding is associated with opposite partial charges attracting with A–B electron cloud repulsion (contractions), whereas chemical bonding involves electron mixing and hybridization (for a more detailed discussion, see ref 26). To classify adsorption configurations as either physisorbed or chemisorbed, we employed an ELF analysis, in which the absence of chemical bonds indicates physical binding. ELF analysis has proven to be highly effective in characterizing bonding nature.^2,26^ This method relies on the system’s equilibrium electron structure only, offering an alternative to more complex techniques like charge difference density (CDD), which can introduce interpretational challenges due to its nonequilibrium components. By assessing electron sharing between the molecule and the substrate, we determine chemisorption, whereas a lack of electron sharing indicates physisorption.

The ELF at the interface of both the physisorbed and chemisorbed configurations is shown in Figure 3. In both cases, it is evident that the aminic nitrogen (NH) atoms do not form chemical bonds with the underlying Ag atoms. However, the interactions of the iminic nitrogen (N) atoms differ significantly. In the physisorbed configuration (Figure 3e), these atoms interact with the substrate through van der Waals forces, whereas, in the chemisorbed configuration (Figure 3b), a clear chemical bond forms between the nitrogen and an Ag atom.

**Figure 3:**
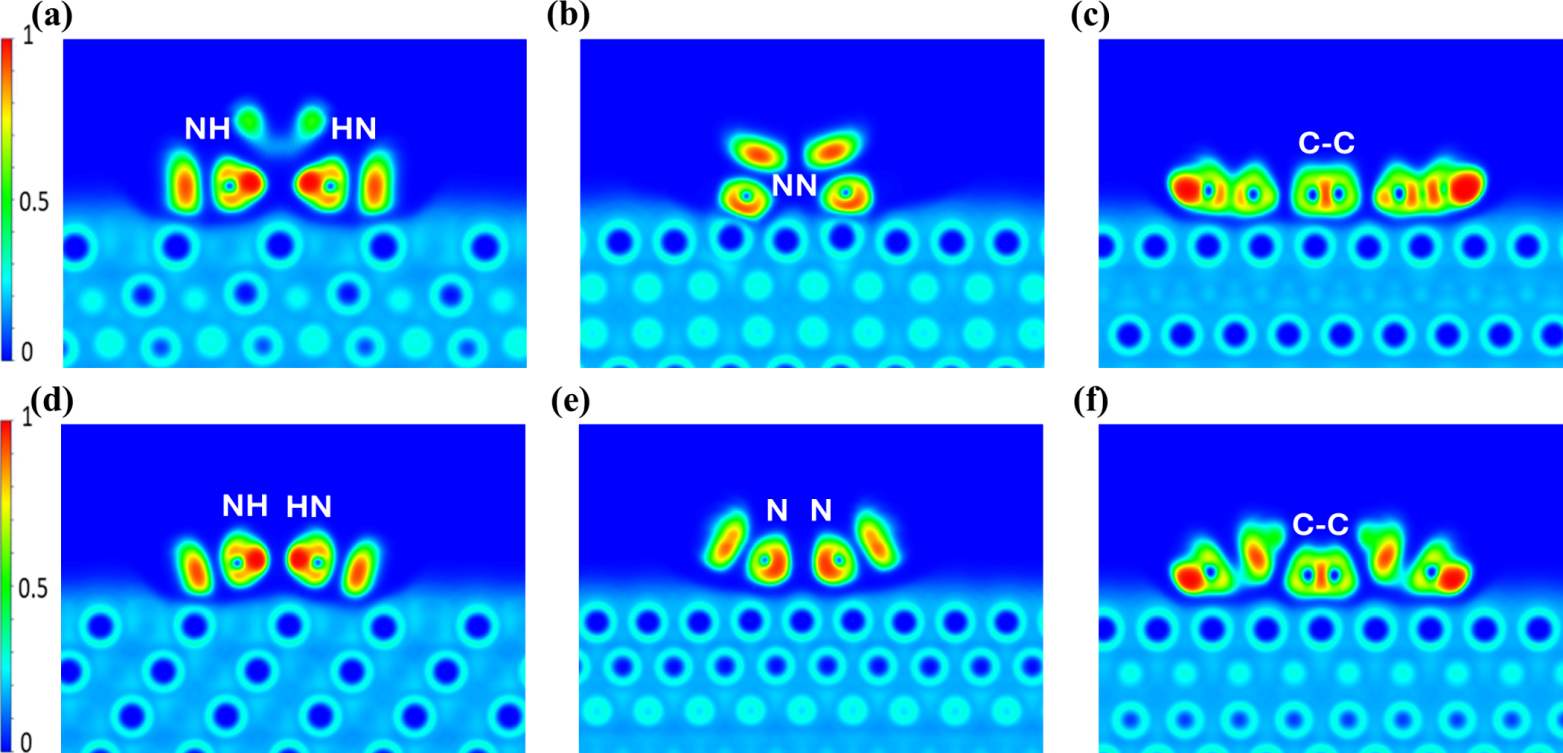
ELF plots along planes passing through the chemisorbed (upper row, a–c) and physisorbed (lower row, d–f) 2*H*-TPP on Ag(111) systems. The planes intersect (a,d) the aminic pyrrole rings, (b,e) the iminic pyrrole rings, and (c,f) the C7–C8 bond of the aminic pyrrole ring (for carbon atom numbering, see Figure 1).

Only in the chemisorbed configuration do we observe electron sharing (indicative of chemical bonding) and hybridized electronic states, demonstrating an interaction between the molecule and the Ag surface. This highlights the fundamental difference in the binding mechanisms of the two configurations. However, note that both configurations exhibit strong attachment to the surface. In fact, the strong physical binding observed for 2*H*-TPP, which does not rely on permanent dipole interactions (Keesom forces), has been reported in only a limited number of cases. Typically, physisorbed molecules are bound more weakly.

The ELF analysis reveals that both configurations exhibit physical binding contributions from the aminic (NH) nitrogens and the phenyl legs. In contrast, the formation of chemical bonds is limited to the iminic nitrogens, which undergo a notable charge redistribution. Accordingly, the designations “physisorbed” and “chemisorbed” are based on these ELF observations. To quantitatively assess the electron transfer between the molecule and the surface, we carried out a Bader charge population analysis.^28^ This analysis uncovers a significant disparity in net charge transfer between the 2*H*-TPP molecule and the Ag(111) surface: chemisorbed 2*H*-TPP has a total charge transfer of −0.93 e from the substrate, while the physisorbed case shows a transfer of −0.07 e. The former is significant and large compared to the Cu(111) surface,^43^ while the latter is negligible as expected. It should also be noted that GGA-HCTH calculation of adsorption of 2*H*-TPP at a step-edge on Ag(111)^68^ indicates that the molecule takes up 0.191e according to Hirshfeld analysis and thus slightly larger than we found for the physisorbed state.

Figure 4 compares the density of states (DOS) of gas-phase 2*H*-TPP with the molecular projected density of states (PDOS) for the adsorbed configurations. A Gaussian smearing of 0.025 eV was applied, and the PDOS was evaluated by using 1000 energy points to ensure adequate spectral resolution. In the gas phase, 2*H*-TPP exhibits sharp, discrete peaks corresponding to well-defined molecular orbitals, including the HOMO and LUMO states.^67^

**Figure 4:**
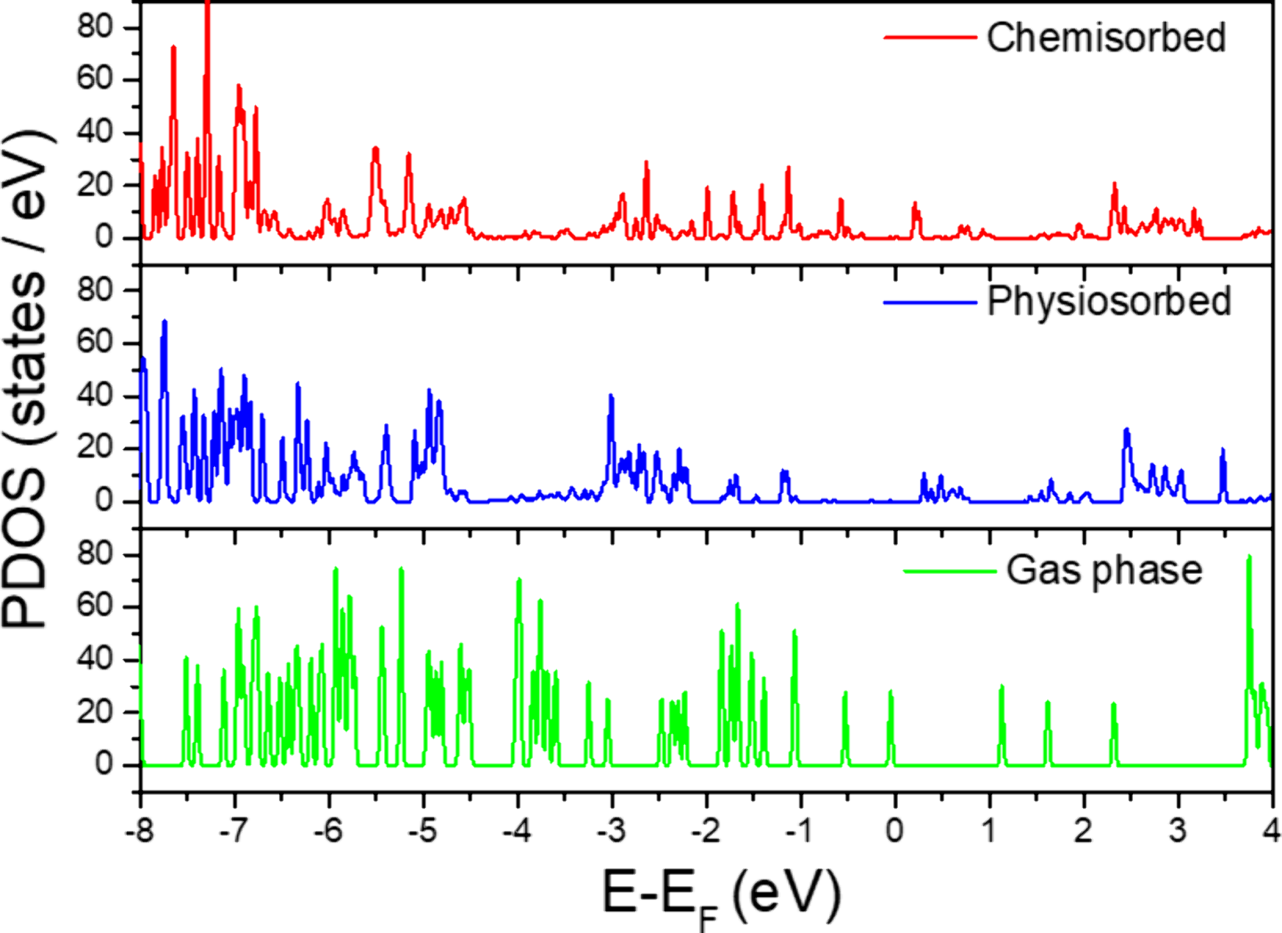
Molecular projected density of states (PDOS) for free 2*H*-TPP (gas phase) compared to 2*H*-TPP absorbed to Ag(111) in chemisorbed and physiosorbed configurations using r^2^SCAN+rVV10. The energy scale is relative to the Fermi energy, *E*_F_.

Upon physisorption on Ag(111), these features broaden due to weak hybridization with Ag d-states, while the overall molecular fingerprint remains largely intact. The HOMO shifts from −0.2 eV in the gas phase to −1.0 eV upon adsorption, and the LUMO moves from ∼1.0 eV toward the Fermi level, resulting in a reduced HOMO–LUMO gap of approximately 1 eV.

More pronounced modifications are observed for the chemisorbed configuration. The occupied PDOS shows clear state splitting and an overall upward shift of ∼0.5 eV relative to the physisorbed case, bringing both frontier orbitals closer to the Fermi level. This shift reflects stronger molecule–substrate interactions and enhanced electronic coupling. A distinct feature appearing at −2.3 eV is attributed to hybridization between the pyrrolic nitrogen orbitals and Ag d-states. Consistently, the PDOS intensity near the Fermi level increases significantly, indicating stronger orbital mixing. These changes arise from adsorption-induced structural distortions and charge transfer between the molecule and substrate, as corroborated by the Bader charge analysis.

This seems to agree with published experimental ultraviolet photoelectron spectroscopy (UPS) and inverse photoemission spectroscopy (IPES) data for 2*H*-TPP adsorbed on coinage-metal surfaces.^68^ For physisorbed 2*H*-TPP on Ag(111), experimental spectra preserve the molecular character with modest broadening, in good agreement with the calculated PDOS. In contrast, the chemisorbed configuration systems exhibit stronger coupling and show an upward shift of the HOMO toward the Fermi level and increased spectral broadening, distinct from experimentally observed spectral trends.^67^ The narrowing of the HOMO–LUMO gap and enhanced spectral weight near *E*_f_ captured in our calculations closely reproduce these experimental observations.

Since the physisorbed configuration is lower in energy than the chemisorbed configuration, the latter might never be realized under normal conditions, possibly only by being pressed against the surface. Nevertheless, by probing the potential energy surface between these two adsorption configurations, the energy barrier can be determined by using the CI-NEB method. This calculation was performed along the shortest reaction path, connecting the stable physisorbed configuration to an adjacent physisorbed configuration through the chemisorbed configuration, as depicted by the purple, yellow and red footprint boxes, respectively, in Figure 5a. For comparison, the reaction path connecting two adjacent most stable physisorbed configurations was also probed, as illustrated with purple and yellow color footprint boxes, as shown in Figure 6a. For these relatively short diffusion paths, seven intermediate images were used to connect the equilibrium configuration with the chemisorption/transition state configuration to simulate the potential energy diffusion paths. The results show a large reaction barrier of 1.2 eV for the transition from physisorption to chemisorption (Figure 5b). This barrier must be considered in the context of the energy difference between the two configurations of 0.95 eV. The relatively large energy barrier together with the considerably higher chemisorption energy indicates that this configuration is unlikely to be reached under normal deposition conditions. Even so, we have found the barrier to go from the chemisorbed configuration back to the equilibrium physisorption to be around 65 meV. In comparison, a barrier of 24 meV is required for diffusion between two adjacent physisorbed configurations (Figure 6b). The barrier between the physisorption sites is large enough for 2*H*-TPP to be immobilized on Ag(111) at room temperature.

**Figure 5:**
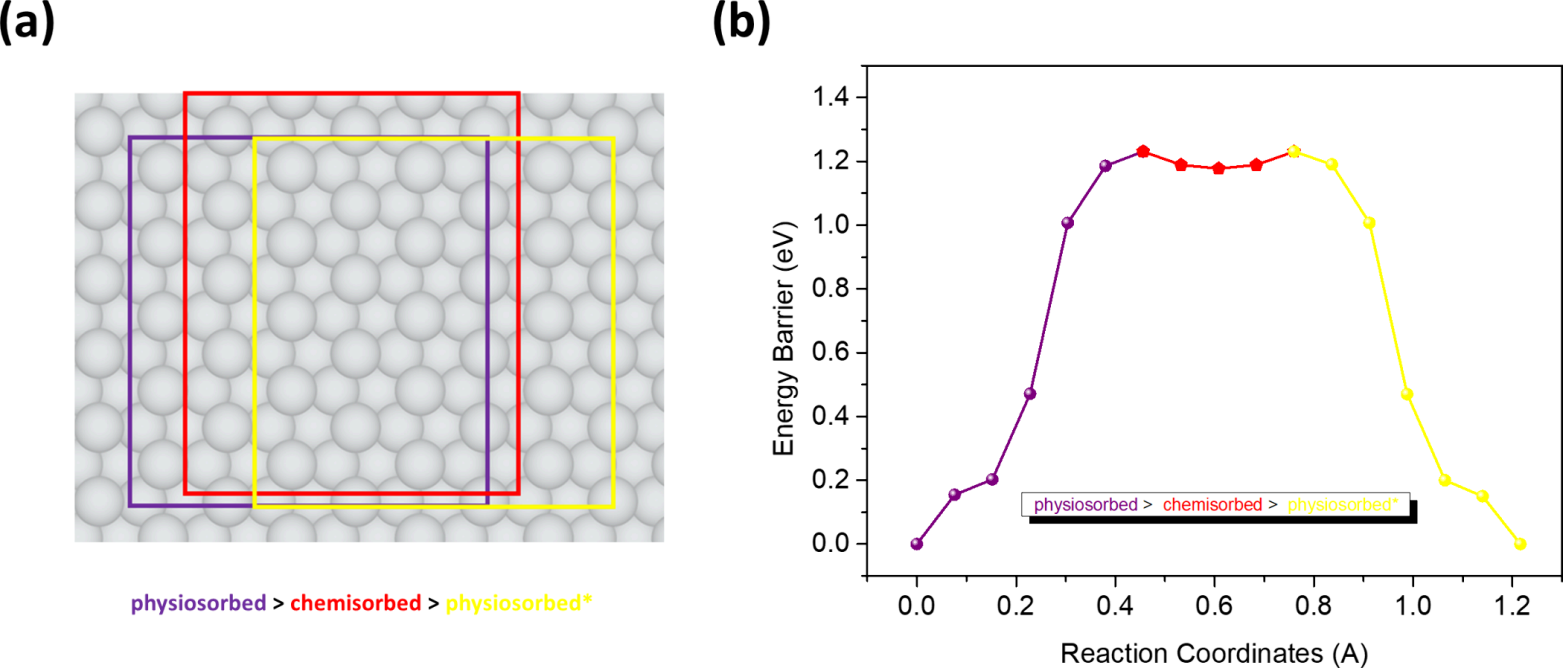
Diffusion of 2*H*-TPP from (a) a physisorbed (purple) to an adjacent physisorbed (yellow) configuration via the intermediate chemisorbed (in red) configuration and (b) potential energy surface as simulated by CI-NEB.

**Figure 6:**
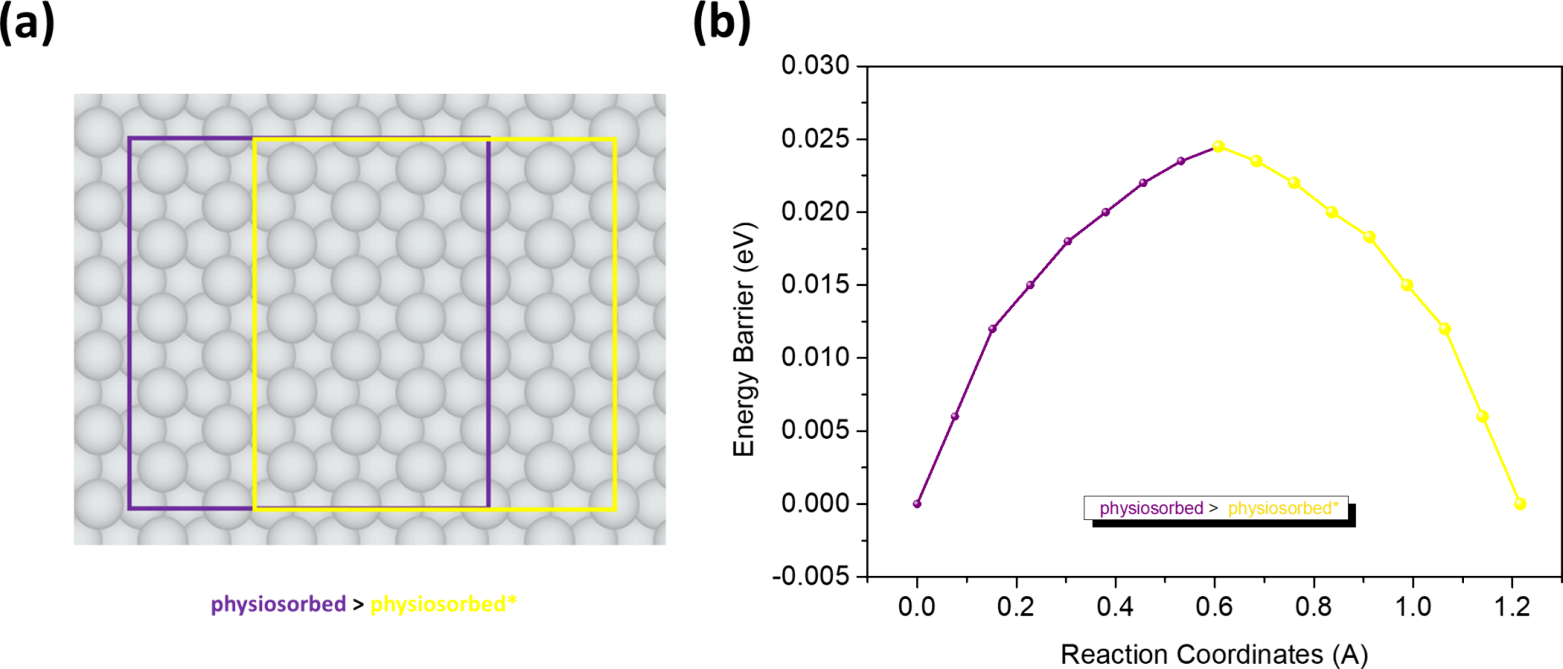
Direct diffusion of 2*H*-TPP between two adjacent (a) physisorbed (purple and yellow) configurations and (b) potential energy surface as simulated by CI-NEB.

Controlling whether a molecule undergoes physisorption or chemisorption is crucial for tailoring interface properties on surfaces.^30,71^ For example, chemical bonding can establish electrical pathways that yield Ohmic contacts between a molecule and its substrate, while physical adsorption typically creates a Schottky barrier due to electron tunneling. Density of states (DOS) for 2*H*-TPP on Ag(111) is quite similar in both adsorption modes. In contrast, the electron density repulsion between the substrate and the molecule in the physisorbed configuration can provide an isolation effect that might extend the spin lifetimes of magnetic molecules. In the case of 2*H*-TPP on Ag(111), the physisorption is sufficiently robust to immobilize the molecule on the surface with an energy barrier of 24 meV separating two neighboring equilibrium configurations.

## 4 Conclusions

We have studied the adsorption of 2*H*-TPP on Ag(111) using the r^2^SCAN-rVV10 functional and have found a stable physisorbed ground state along with a metastable chemisorbed configuration with considerably higher energy. The adsorption configurations were also validated using PBE-D3 and optB86b-vDWDF2. The physisorbed configuration is 0.95 eV lower in energy than the chemisorbed configuration. Their binding energies to the silver substrate are estimated to be −6.35 and −5.40 eV, respectively. The energy reduction in creating covalent bonding associated with chemisorption is counteracted by the increase in energy by molecule deformation. Their mode of binding has been determined using the electron localization function (ELF), which offers a straightforward way to differentiate between strong physical and weak chemical bonding. The chemisorbed configuration has an average height 0.576 Å lower than that of the physisorbed one. The Bader charge population analysis has revealed that there is a negligible charge transfer from the surface for the physisorbed configuration and a quite considerable charge transfer for the chemisorbed configuration. These interface interactions and accompanying molecular distortions significantly alter the position and shape of the molecular states as confirmed by our PDOS results. Hence, the overall conclusion from the Bader charge, ELF, and DOS analyses is that physisorption, particularly in large and flexible molecules, can induce large shifts in the DOS, typically associated with chemisorption. This kind of strong physisorption could easily be misinterpreted as if chemical bonds have been formed between the molecule and the substrate in experimental measurements of properties that are DOS-dependent.

## Supplementary Material



## Notes

Optimized adsorption geometries, bond length comparisons, adsorption heights and distances, ELF-profile plots, and molecular projected density of states (PDF)
