# Supplementary material for: Density-Functional Theory Shows 2H‑Tetraphenylporphyrin Prefers Physisorption over Chemical Bonding on Ag(111)
Source: ACS Omega. 2026 Mar 29;11(13):20616–25. doi: 10.1021/acsomega.5c12411 (PMC13063048; doi:10.1021/acsomega.5c12411)
Supplement: Supplementary file 1 [file ao5c12411_si_001.pdf]

**Supplementary Information**

# Density-Functional Theory Shows 2H-Tetraphenylporphyrin Prefers Physisorption Over Chemical Bonding on Ag(111)

Shabnam Naseri[a]*, Gustav Johansson[a], Ghulam Abbas[a,b], Muhammad Sajjad[c], and J. Andreas Larsson[a,d]

[a]Luleå University of Technology, Department of Engineering Sciences and Mathematics, Laboratorievägen 14, SE-971 87, Luleå, Sweden

[b]Linköping University, Department of Physics, Chemistry and Biology, SE-581 83, Linköping, Sweden

[c]Nottingham Ningbo China Beacons of Excellence Research and Innovation Institute, University of Nottingham Ningbo China, Ningbo, Zhejiang 315100, China

[d]Luleå University of Technology, Wallenberg Initiative Materials Science for Sustainability, Laboratorievägen 14, SE-971 87, Luleå, Sweden

E-mail: Shabnam.naseri@associated.ltu.se

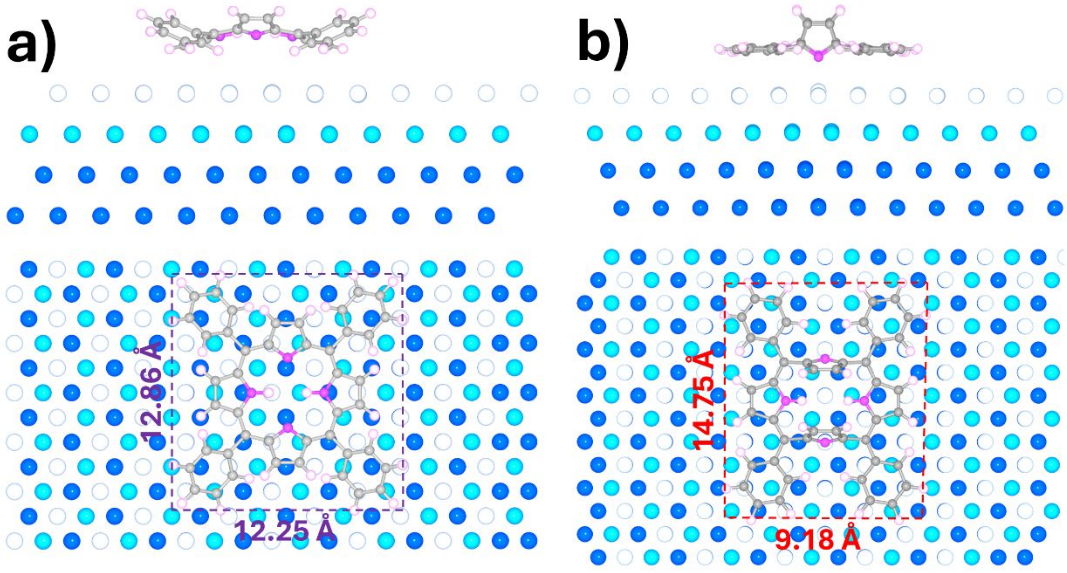

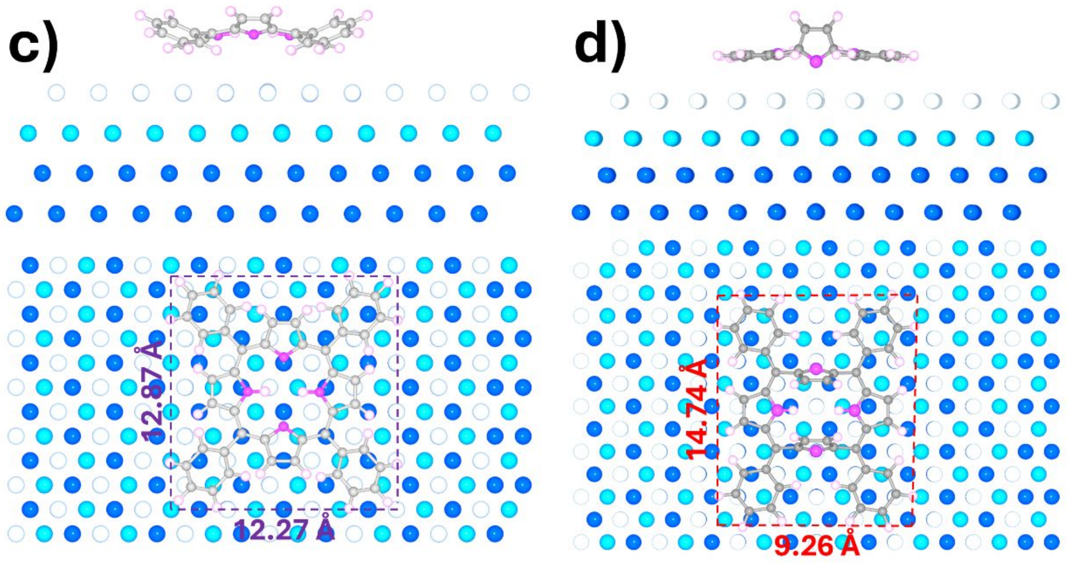


Figure S1. The optB86b-vDWDF2 relaxed geometries of 2H-TPP on the Ag(111) surface: a) physisorbed 2H-TPP, and b) chemisorbed 2H-TPP, and the PBE-D3 relaxed geometries of 2H-TPP on the Ag(111) surface: c) physisorbed 2H-TPP, and d) chemisorbed 2H-TPP in side- and top views, respectively. The light to dark shade of blue is used to represent the 1st, 2nd, and 3rd layer of Ag(111). The gray, purple, and white represent C, N, and H respectively.

Table S1. Comparison of bond lengths (in Å) of 2H-TPP in gas phase, and when either physisorbed, chemisorbed on Ag(111)., The calculations are performed using r2SCAN+rvvw10 (optB86b-vdW, PBE-D3) functional. The numbering of atoms is according to Figure 1.

| Bond | Free molecule | Physisorbed | Chemisorbed |
|---|---|---|---|
| C1−H1 | 1.091 (1.092, 1.085) | 1.091 (1.093, 1.085) | 1.090 (1.093, 1.085) |
| C1−C2 | 1.398 (1.397, 1.391) | 1.397 (1.398, 1.392) | 1.401 (1.401, 1.395) |
| C2−H2 | 1.091 (1.093, 1.085) | 1.091 (1.093, 1.085) | 1.091 (1.094, 1.086) |
| C2−C3 | 1.394 (1.394, 1.389) | 1.395 (1.396, 1.390) | 1.398 (1.399, 1.394) |
| C3−H3 | 1.091 (1.093, 1.085) | 1.090 (1.092, 1.084) | 1.085 (1.087, 1.079) |
| C3−C4 | 1.410 (1.409, 1.405) | 1.410 (1.410, 1.405) | 1.420 (1.421, 1.417) |
| C4−C5 | 1.474 (1.474, 1.470) | 1.482 (1.483, 1.479) | 1.461 (1.465, 1.460) |
| C5−C9 | 1.438 (1.437, 1.433) | 1.419 (1.420, 1.414) | 1.485 (1.489, 1.487) |
| C9−C10 | 1.452 (1.451, 1.446) | 1.456 (1.456, 1.450) | 1.405 (1.403, 1.395) |
| C10−H6 | 1.087 (1.089, 1.082) | 1.085 (1.087, 1.079) | 1.087 (1.090, 1.082) |
| C9−N2 | 1.368 (1.368, 1.363) | 1.374 (1.374, 1.369) | 1.388 (1.388, 1.386) |

| | | | |
|---|---|---|---|
| C5−C6 | 1.417 (1.415, 1.412) | 1.414 (1.414, 1.409) | 1.411 (1.413, 1.410) |
| N1−H5 | 1.018 (1.020, 1.011) | 1.021 (1.023, 1.014) | 1.017 (1.021, 1.013) |
| C6–N1 | 1.381 (1.382, 1.378) | 1.379 (1.380, 1.375) | 1.383 (1.382, 1.377) |
| C6–C7 | 1.439 (1.438, 1.433) | 1.435 (1.434, 1.429) | 1.453 (1.454, 1.449) |
| C7–H4 | 1.084 (1.086, 1.078) | 1.084 (1.086, 1.079) | 1.083 (1.085, 1.077) |
| C7–C8 | 1.380 (1.380, 1.374) | 1.384 (1.384, 1.377) | 1.382 (1.382, 1.377) |

Table S2. Heights and the shortest distances to Ag for 2H-TPP physisorbed or chemisorbed on Ag(111) in Å. $\underline{h}$(C/N) is the average height of the C and N atoms to the Ag surface, $h$(C) and $h$(N) are the minimum heights of a C atom and N atom respectively to the Ag surface; $d$(C) and $d$(N) are the shortest distances between a Ag atom and a C and N atom respectively.

| | **$\underline{h}$(C/N)** | **d(C)** | **d(N)** | | **h(C)** | **h(N)** | |
|---|---|---|---|---|---|---|---|
| | | | **aminic** | **iminic** | | **aminic** | **iminic** |
| PBE-D3 (physisorbed) | 3.455 | 3.027 | 3.576 | 3.499 | 2.851 | 3.352 | 3.356 |
| optB86b (physisorbed) | 3.500 | 2.913 | 3.529 | 3.627 | 2.828 | 3.325 | 3.402 |
| $r^2$SCAN+rvv10 (physisorbed) | 3.441 | 3.126 | 3.613 | 3.471 | 2.831 | 3.306 | 3.314 |
| PBE-D3 (chemisorbed) | 2.980 | 2.860 | 3.208 | 2.273 | 2.795 | 2.976 | 2.223 |
| optB86b (chemisorbed) | 2.905 | 2.808 | 3.133 | 2.255 | 2.748 | 2.919 | 2.181 |
| $r^2$SCAN+rvv10 (chemisorbed) | 2.865 | 2.783 | 3.043 | 2.283 | 2.741 | 2.907 | 2.134 |

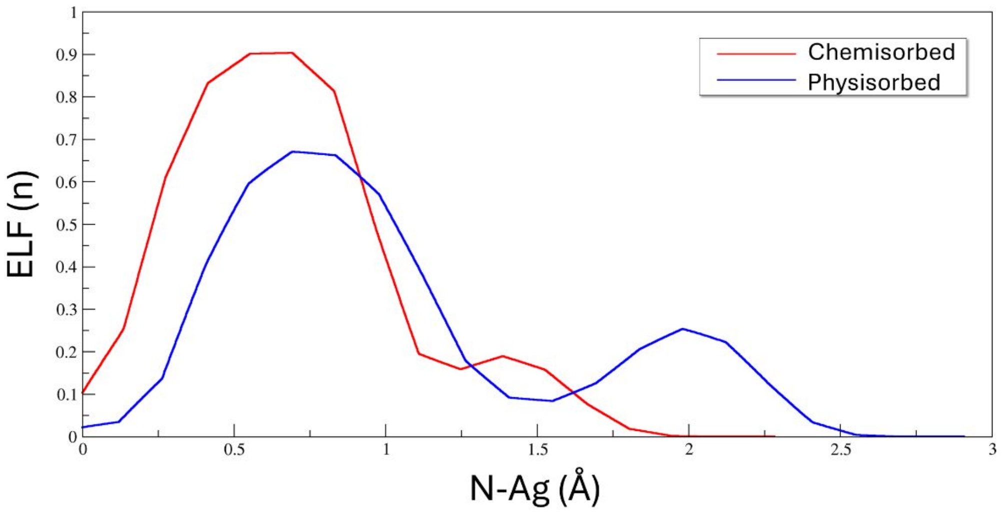


Figure S2. ELF-profiles plots of the chemisorbed and physisorbed configuration of 2H-TPP on Ag(111) between a pyrrole carbon and Silver atom from the center of the N-atom core at 0 Å to the Ag core at 2.30 Å (chemisorbed configuration) and 2.90 Å (physisorbed configuration).

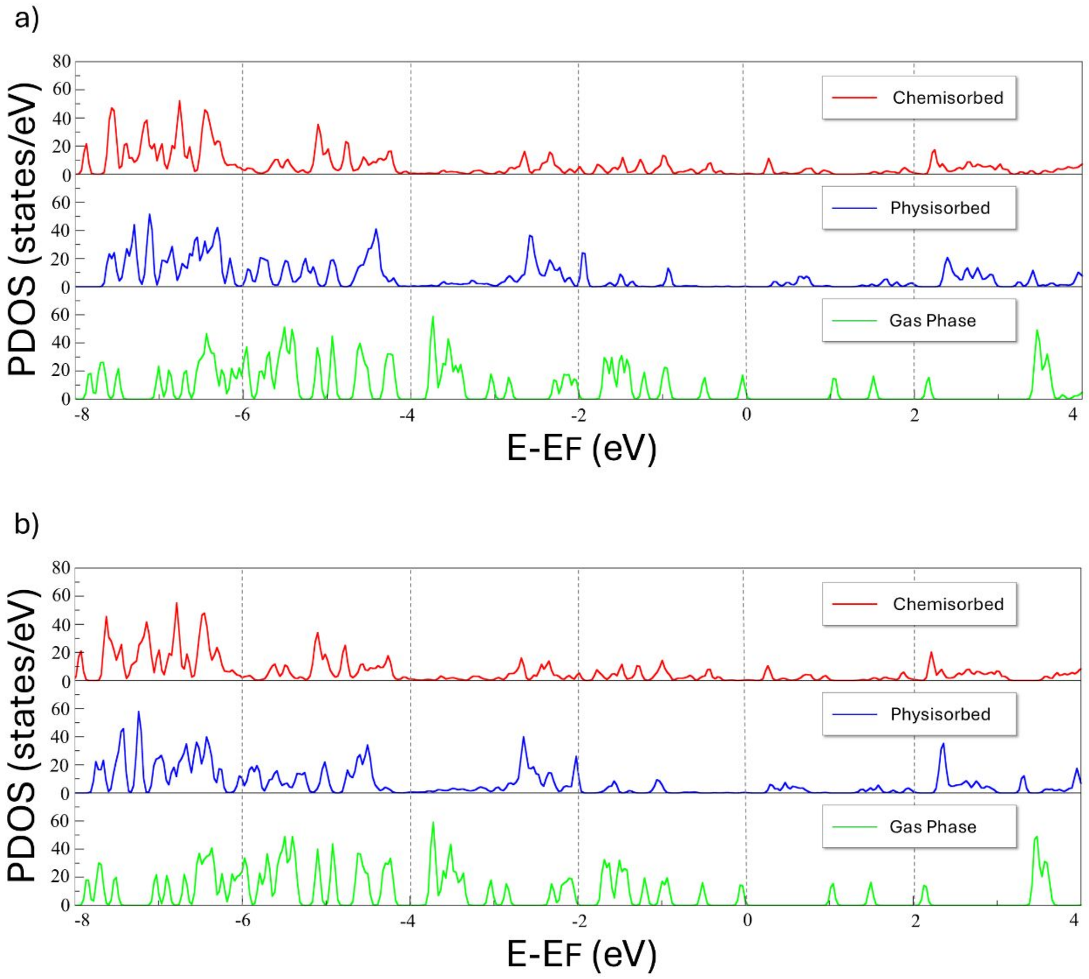

Figure S3. The molecular projected density of states (PDOS) for free 2H-TPP (gas phase) compared to 2H-TPP@Ag(111) in chemisorbed and physiosorbed configuration using a) optB86b-vdW, and b) PBE-D3. The energy scale is relative to the Fermi energy.
